# Use of Palliative Cisplatinum for Advanced Cervical Cancer in a Resource-Poor Setting: A Case Series From Kenya

**DOI:** 10.1200/JGO.2016.006411

**Published:** 2016-11-02

**Authors:** Elkanah Orang’o, Peter Itsura, Philip Tonui, Hellen Muliro, Barry Rosen, Luc van Lonkhuijzen

**Affiliations:** **Elkanah Orang’o**, **Peter Itsura**, **Philip Tonui**, and **Hellen Muliro**, Moi University School of Medicine, Eldoret, Kenya; **Barry Rosen**, Oakland University, Rochester, MI; **Luc van Lonkhuijzen**, Academic Medical Center, Amsterdam, the Netherlands.

## Abstract

**Purpose:**

To evaluate the effectiveness and feasibility of cisplatinum for palliative treatment of advanced cervical cancer in a resource-poor setting.

**Methods:**

An observational case series is reported from a university teaching hospital in Kenya. All women presenting with advanced cervical cancer and planned for palliative cisplatinum therapy from 2010 to 2014 were included. Women were treated with cisplatinum 50 mg/m^2^ every 4 weeks in an outpatient setting. Data on tumor stage and symptoms control were prospectively collected in an electronic database. The main outcome measure was control of symptoms such as bleeding, discharge, and pain.

**Results:**

Of the women who originally presented with bleeding, 62% reported improvement in this symptom, 31.3% reported the bleeding completely stopped, 58% had improvement of their vaginal discharge, and 20.5% reported complete resolution. Of the women who presented with pain, 54% reported improvement; 30.9% reported pain had completely resolved. After each treatment cycle, approximately 30% of patients did not return for their next treatment.

**Conclusion:**

Cisplatinum as palliative treatment of advanced cervical cancer is feasible in a resource-poor setting and leads to effective symptom control. However, unknown barriers may inhibit women from returning for regular treatment.

## INTRODUCTION

In Kenya and other parts of sub-Saharan Africa, most women with cervical cancer present with an advanced stage of the disease. In 2012, the WHO stated that there were 528,000 new cervical cancer cases annually worldwide, of which 84% occurred in the less-developed world.^1^ Cervical cancer is the most common gynecologic cancer in Kenya and other Eastern African countries. Mortality in high-resource countries is 4.1 women per 100,000, whereas it is five times greater in Kenya (21.8 per 100,000 [age-standardized rates]). It is possible that the mortality is even higher than reported because the quality of the data for cancer in Kenya and surrounding countries is limited and likely misses a proportion of the cervical cancer cases. In Kenya, cervical cancer is the leading cause of cancer mortality.^1^

Many factors contribute to the difference in cervical cancer burden (ie, prevalence and mortality) between high- and low-resource countries, including lack of comprehensive screening, lack of adequate treatment, and barriers to access to health care. In most regions of the world, women with advanced cervical cancer would be treated with a combination of chemotherapy and radiotherapy. Chance of cure would range from 30% to 40%, but the likelihood of benefiting and getting relief of symptoms would be extremely high, greater than 95%. In Kenya, there is limited access to both chemotherapy and radiotherapy. For a woman to receive radiotherapy treatment in Kenya, she would need to travel to Nairobi. This is a 6-hour drive from our hospital in Eldoret. She would also have to arrange and pay for her lodging and, even in that situation, she would have to wait between 6 and 12 months before starting treatment. Only external beam therapy is available in Nairobi; the cost is KES 80,000 (US $785). An alternative option would be to travel to Kampala, Uganda, also a 6-hour drive, where both external beam and intracavitary treatment are available at a cost of KES 160,000 (US $1,570).

In Kenya, 79% of the population live in a rural setting, and 33.6% live below the poverty line, defined as living on less than $1.90/day. Kenya is also one of the African countries hardest hit by HIV/AIDS. Fifteen percent of women diagnosed with cervical cancer are HIV positive.^2^ Most women live in rural settings and below the economic poverty line. For the majority of those women diagnosed with cervical cancer, radiotherapy is simply not accessible.

In the neoadjuvant setting, cisplatinum in doublet or triplet combination with other chemotherapeutics in intervals of 10-20 days for three cycles has been shown to lead to complete or partial tumor response in more than half of women.^3^ Basile et al^4^ suggested the use of platinum-based chemotherapy for palliative treatment of advanced or recurrent cervical cancer when radiotherapy facilities are far away, as it is in many African countries.^4^ In Brazil, a retrospective study of 153 women treated with carboplatin and paclitaxel for advanced and recurrent cervical cancer showed that 34.6% had an objective response and a median survival of 10.6 months.^5^ In Canada, 40% of women (n = 25), most of whom were pretreated with radiation, had a complete or partial response after palliative treatment with carboplatin and paclitaxel; the median overall survival was 21 months.^6^ In Nigeria, administration of cisplatin 70 mg/m^2^ every 3 weeks resulted in cessation of vaginal bleeding for 81 of 116 patients.^7^

Our hypothesis was that for women who could not access radiotherapy, single-agent cisplatinum would alleviate symptoms in those presenting with advanced cervical cancer. Our objectives were to assess the feasibility and acceptability of single-agent cisplatinum for palliative symptom control of advanced cervical cancer in a low-resource setting.

## METHODS

We conducted this study at Moi Teaching and Referral Hospital, in Eldoret in western Kenya. This is a level 6 referral hospital that serves a population of 5 million people. It recently developed an oncology program in collaboration with AMPATH (Academic Model Providing Access to Healthcare), a consortium of North American universities led by Indiana University. The hospital recently built a new outpatient facility to see and treat oncology patients. It does not yet have the capacity to provide radiation therapy.

All women who presented with cervical cancer between January 2010 and December 2014 were evaluated in a gynecologic oncology clinic setting. For every woman, demographics and disease-related data were captured in a prospective electronic database. For this study, we included all women who planned to undergo chemotherapy treatment with palliative intent and who received at least one cycle of cisplatinum.

Chemotherapy consisted of cisplatinum 50 mg/m^2^ every 4 weeks. This is a lower dose and frequency than is used in the neoadjuvant setting, where the aim is to reduce tumor size to make patients amenable for surgery. In an early Gynecologic Oncology Group study evaluating different cisplatinum doses, patients receiving 100 mg/m^2^ every 21 days had better response rates but no improvement in complete remission rate, response duration, progression-free interval, and survival as compared with those receiving 50 mg/m^2^ every 21 days.^8^ However, this higher dose was also associated with greater myelosuppression and nephrotoxicity.^8^ In our situation, cisplatinum was used with palliative intent and aimed at symptom control. By reducing the dose and frequency, we wanted to make the treatment as tolerable for these women as possible. The treatment regimen included hydration with 1 L of intravenous normal saline before and after chemotherapy. Premedication also included intravenous ondansetron 8 mg and dexamethasone 12 mg administered 30 minutes before administration of cisplatinum. Postchemotherapy, the patients were observed for at least 1 hour. On discharge, ondansetron 4 mg was continued twice daily for a week and women were advised to increase oral fluid intake to at least 2 L/day. If, during a subsequent cycle, a patient reported excessive nausea and vomiting during the previous chemotherapy session, she was advised to stay overnight in the hospital for parenteral rehydration and antiemetics (eg, ondansetron, dexamethasone, and even, sometimes, parenteral chlorpromazine). When women presented with pain, morphine syrup was used to support pain control. However, morphine was not expected to control bleeding or discharge. No hemostatic agents were given and only if women had a low hemoglobin level and presented with complaints was a blood transfusion offered. Chemotherapy was prepared in the dedicated pharmacy and delivered by nurses in an outpatient day-care setting. Chemotherapy was continued until a maximum of six doses or control of symptoms had been achieved.

Before the first and each subsequent cycle of chemotherapy, patients had complete blood cell count and serum creatinine measurements. Cisplatinum was postponed as long as the hemoglobin level was < 8 mg/dL, the creatinine level was > 80 mmol/L, and the absolute neutrophil count was < 0.001/μL.

The cost for treatment was KES 3,930 (US $40), which included consultation, administrative cost, and chemotherapy. Initially, when the program was subsidized by AMPATH, the patient cost was only KES 400 (US $4). Currently, a financial support program is available to assist those who can only afford a partial payment.

At every follow-up visit, patients reported their symptoms and whether the symptoms were the same, better, or worse. To establish the rate at which symptoms improved, we also included those women in the denominator who only came once and for whom we could not evaluate the effect of chemotherapy. Rates reported are for the entire cohort. If women had not returned for follow-up, their relatives were contacted by telephone in May 2015. In this way, disease status and, if applicable, date of death was ascertained. Adverse effects of treatment were not recorded systematically.

Data are summarized using descriptive statistics. The Moi University/Moi Teaching and Referral Hospital Institutional Research and Ethics Committee approved this study. All women gave informed consent.

## RESULTS

Between January 2010 and December 2014, 519 women were diagnosed with cervical cancer in our hospital. Of these, 106 (20%) were planned to receive neoadjuvant chemotherapy followed by surgery, 90 were planned to undergo radical hysterectomy, 67 (13%) were referred for radiotherapy, and 44 (9%) were referred for palliative hospice care. After evaluation, and discussion with the patient and relatives about possible options, 124 women (24%) were planned to receive palliative chemotherapy. These were women who were not able to afford the cost or had other reasons to refuse radiotherapy. Not all of these women went on to have chemotherapy, because some had renal impairment that prevented the use of cisplatinum and others did not return for their planned chemotherapy. Of the 124 women, 98 went on to have at least one cycle of chemotherapy and were included for this study. The mean age of this cohort was 53 years (range, 29-81 years). Eighty-five percent of the patients were International Federation of Gynecology and Obstetrics stage 3 or 4. Of the 77 patients tested, 25 (32.4%) were HIV positive and a CD4 cell count was known for 13 of these (mean, 472 CD4 cells; range, 34-732 CD4 cells). All women were already receiving antiretroviral treatment or started this treatment as soon as the diagnosis of cervical cancer had been made. Forty-five percent of the patients undergoing treatment received three or more cycles of chemotherapy (Table 1).

**Table 1:**
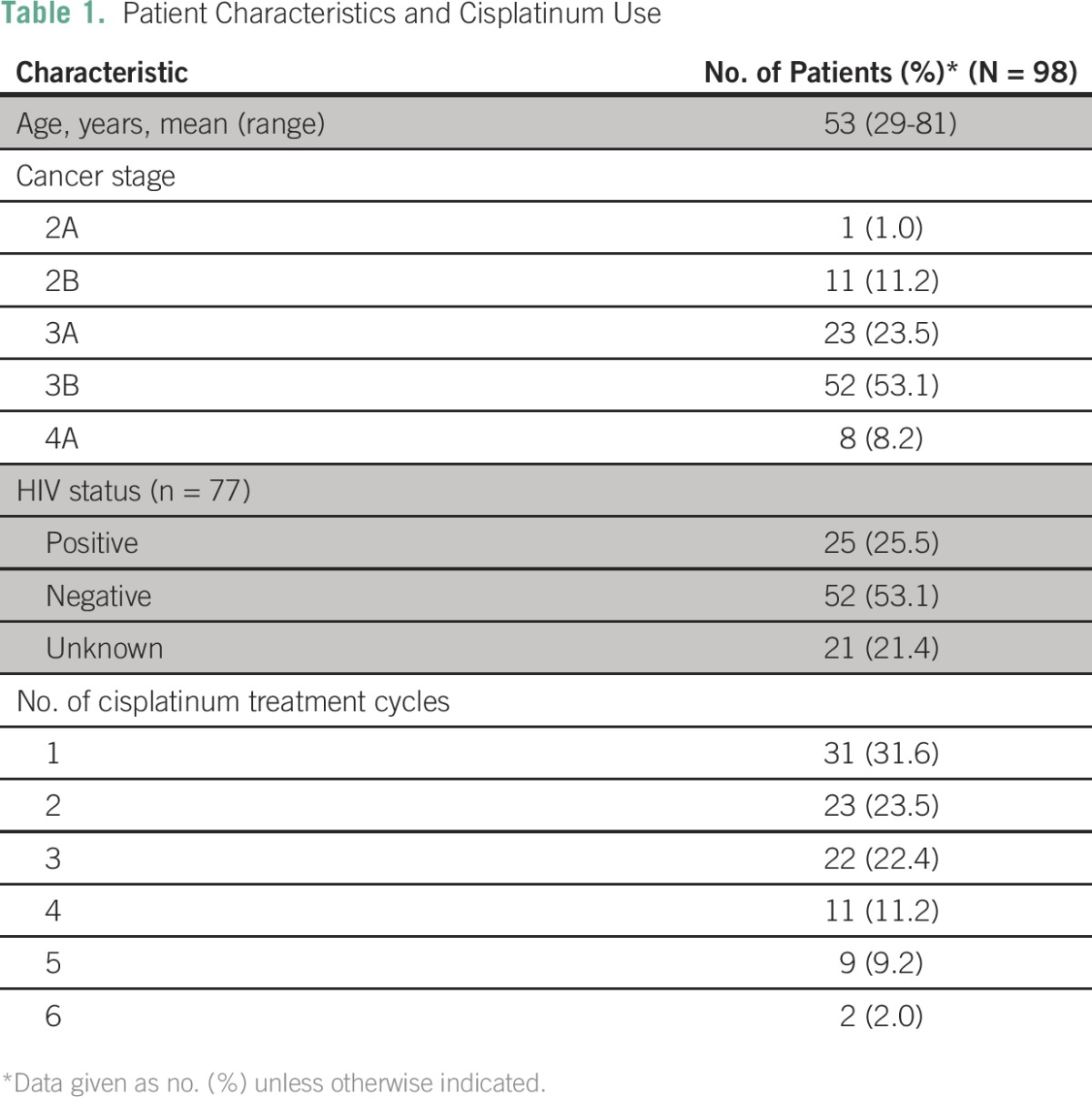
Patient Characteristics and Cisplatinum Use

Most women came to the hospital because of their symptoms. We identified less than 20% through our cervical cancer screening program. Of the women in our study, 83 (84.6%) presented with bleeding, 68 (69.3%) with discharge, and 68 (69.3%) with low abdominal pain.

**Fig 1:**
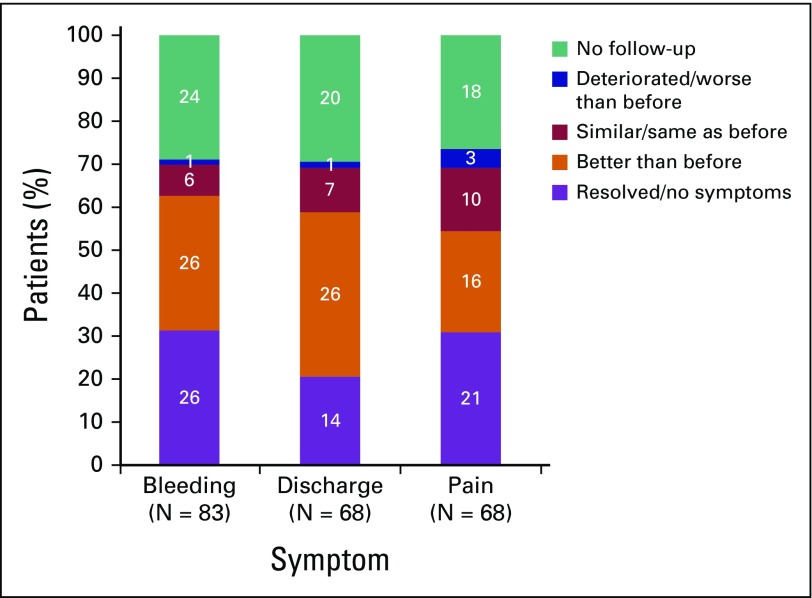
Palliative cisplatinum for advanced cervical cancer in Kenya. Response to treatment.

At each subsequent visit after the first cycle of cisplatinum, the response of the symptoms to treatment was evaluated. These data are presented in Figure 1. Data on symptom control after one treatment were available for 70% of the patients. The other 30% did not return after their first cycle and response could not be evaluated. Of the women who originally presented with bleeding, 62% reported improvement; for 31.3%, the bleeding completely stopped. Improvement in vaginal discharge was reported by 58% of women; 20.5% reported complete resolution. Of the women who presented with pain, 54% reported improvement; for 30.9%, the pain completely resolved. Symptoms improved after a median of two cycles.

The most common observed chemotherapy adverse effects included nausea and vomiting, nephrotoxicity, abnormal blood parameters (ie, low hemoglobin levels and low absolute neutrophil counts), and peripheral neuropathy. The majority of the women (64.3%) had more than one chemotherapy treatment and we evaluated renal toxicity based on consecutive serum creatinine values after chemotherapy. At the start of their chemotherapy, 85 of 98 women (86.7%) had a normal creatinine level. Of those who had at least two chemotherapy treatments, the creatinine level rose in four of 57 (7%) who initially presented with a normal value. Treatment was never withheld because of adverse effects other than those discussed in the Methods.

## DISCUSSION

Our results show that palliative chemotherapy with single-agent cisplatinum is feasible in a low-resource setting. For the more than half of the women in this study, this treatment improved the most distressing and limiting symptoms, including bleeding, pain, and the foul discharge.

The main limitation to our study is the lack of follow-up, which leads to uncertainty about the length of time symptom control is maintained. One of the possible reasons that women did not return may have been a worsening of symptoms or the adverse effects the women experienced. However, there are many other barriers that prevent these women from attending our clinic and receiving treatment. One-fifth of women never even started the proposed treatment, which not only affected our study population but was also true for all women with cervical cancer seen in this period. Lack of funds to cover both the direct and indirect costs of those seeking treatment in the hospital probably played a significant role in limiting their ability to access treatment. Some women choose to use indigenous medicine, some do not have independent decision-making power in their home, some have transportation difficulties, and some a lack of understanding of their cancer, all of which may play a role. These barriers, in combination with barriers in the health-care system, are probably also the reason so many women present so late with their disease.^9^

The generalizability of our study is limited to a specific setting. We have shown that delivery of chemotherapy is safe and can be done in a low-resource setting, but it does require specific infrastructure. Our hospital includes a pharmacist, trained nursing staff, and trained physicians, and a supply chain that ensures that drugs and materials needed to administer these are routinely available. This means chemotherapy will likely have to be administered in a regional hospital and, when not possible, will at least have to be supported by a regional hospital.

We do think that our approach can benefit many women in sub-Saharan Africa. Before initiating this program, women with advanced cervical cancer typically would present to the emergency room because of bleeding, pain, and with or without discharge. They did not know their diagnosis and most often they would have very low hemoglobin levels. They would then be admitted, receive a transfusion of red blood cells, and then be discharged to their homes where they would die. It is highly unlikely that they would have any relief of their symptoms. We found several reports describing cohorts of women such as ours. Some describe women being referred for radiotherapy but do not state whether these women actually got the treatment.^10-12^ Strategies to develop palliation for advanced cervical cancer are necessary for settings where there is limited access to health care, limited expertise about cancer, and no radiation oncology treatment options. This situation defines much of sub-Saharan Africa outside of South Africa.^13^

It is clear that the use of cisplatinum as palliative treatment is not ideal. Treatment with a combination of chemotherapy and radiation would be more effective; however, even a recent Cochrane review did not find any randomized studies to support this.^14^ Also, carboplatinum is an option that is associated with less toxicity, but this is more expensive, which would put it out of reach for most women. Many women with advanced cervical cancer come from regions where there is limited access to medical care and no access to radiotherapy. This study showed that cisplatinum used in this setting did alleviate some of the severe symptoms affecting women with advanced stage cervical cancer.

Improving symptoms also provides an opportunity for these women to have some symptom-free time, to be at home, and to plan for their deaths. Many of these women will have young children and the additional time they have and, in particular, symptom-free time, can be very beneficial to them.

Better even than treating the disease would be preventing it either through vaccination or screening. This palliative program was established in parallel with a cervical cancer screening program that, in turn, is identifying preinvasive disease and early-stage cancers that are amenable to a curative treatment strategy.

Improving care for women with advanced cervical cancer in sub-Saharan Africa is possible despite the multitude of barriers. A potential benefit of this program may be the stimulation of greater interest by governments or regions in starting screening programs once they better understand the impact of advanced cervical cancer on affected women. The alternative of no treatment, no attempt at palliation, is not an acceptable option.
